# Efficacy of a Preparation Based on Calcium Butyrate, *Bifidobacterium bifidum*, *Bifidobacterium lactis*, and Fructooligosaccharides in the Prevention of Relapse in Ulcerative Colitis: A Prospective Observational Study

**DOI:** 10.3390/jcm10214961

**Published:** 2021-10-26

**Authors:** Gian Paolo Caviglia, Federico De Blasio, Marta Vernero, Angelo Armandi, Chiara Rosso, Giorgio Maria Saracco, Elisabetta Bugianesi, Marco Astegiano, Davide Giuseppe Ribaldone

**Affiliations:** 1 Department of Medical Sciences, University of Turin, 10124 Turin, Italy; gianpaolo.caviglia@unito.it (G.P.C.); angelo.armandi@unito.it (A.A.); giorgiomaria.saracco@unito.it (G.M.S.); elisabetta.bugianesi@unito.it (E.B.); 2 Clinic of Gastroenterology, Department of Gastroenterology and Transplantation, Università Politecnica delle Marche, Ospedali Riuniti di Ancona, 60126 Ancona, Italy; deblasio.fe@gmail.com; 3 Department of Internal Medicine, San Matteo Hospital, 27100 Pavia, Italy; marta.vernero@gmail.com; 4 Unit of Gastroenterology, Città della Salute e della Scienza di Torino-Molinette Hospital, 10126 Turin, Italy; mastegiano@cittadellasalute.to.it

**Keywords:** complementary therapy, inflammatory bowel diseases (IBD), maintaining therapy, mesalamine, mesalazine, short chain fatty acids (SCFAs)

## Abstract

Several compounds based on short chain fatty acids and/or probiotics/prebiotics have shown promising results in the therapy of ulcerative colitis (UC), possibly due to its key role in restoring gut homeostasis as well as intestinal barrier integrity. Here, we investigated the efficacy of a patented preparation based on calcium butyrate, *Bifidobacterium bifidum*, *Bifidobacterium lactis*, and fructooligosaccharides (FEEDColon^®^, Princeps, Cuneo, Italy) in maintaining remission and improving subjective symptoms and inflammatory indices in patients with UC receiving 5-ASA therapy. A total of 42 patients were prospectively recruited and randomized in 21 patients receiving combination therapy with mesalamine (5-ASA) plus FEEDColon^®^ and 21 patients treated with standard 5-ASA therapy. Patients were assessed at baseline, at 6-month, and 12-month follow-up (FU). Therapeutic success (defined as Mayo partial score ≤ 2 and faecal calprotectin (FC) < 250 µg/g at 12-month FU) was reached by 32 (76%) patients: 20 (95%) among those treated with 5-ASA + FeedColon^®^, and 12 (57%) among those treated with 5-ASA only (*p* = 0.009). Consistently, patients treated with combination therapy improved subjective symptoms (quality of life, abdominal pain, and stool consistency) and reduced FC values, while those treated with 5-ASA alone, improved neither subjective symptoms nor FC during the FU. In conclusion, FEEDColon^®^ supplementation appears to be a valid add-on therapy for the maintenance of remission in patients with UC. Further multicentre, placebo-controlled, double-blind clinical trials are needed to validate our results on larger cohorts of patients with UC.

## 1. Introduction

Inflammatory bowel diseases (IBD) include Crohn’s disease (CD) and ulcerative colitis (UC), both characterized by a chronic–remittent clinical course [1]. Compared to CD that can occur in any portion of the gastrointestinal tract, UC is limited to the colon [2]. The aetiology and pathogenesis of IBD are not fully understood; it is likely that different genetic and environmental factors [3,4,5,6], associated to an impaired intestinal permeability [7], are involved in the onset and progression of the disease. Furthermore, gut microbiota and its interaction with the intestinal immune system may play a central role in triggering and exacerbating IBD [8,9].

Among the therapeutic armamentarium for the treatment of IBD, mesalamine (5-ASA) is the first-line treatment for patients with mild-to-moderate UC; several clinical trials have confirmed its efficacy in terms of clinical response (response rate up to 80%) and disease remission maintenance (remission rate: 40–70%) in such patients [10,11]. However, a not negligible proportion of patients with UC under mesalamine treatment fails to achieve a durable disease control.

Short chain fatty acids (SCFAs) such as butyrate, propionate, and acetate, are a group of fatty acids with less than six carbons produced by the gut microbiota from the fermentation of dietary substrates [12]. In particular, butyrate represents an important energy source for intestinal epithelial cells and plays a crucial role in maintaining mucosal homeostasis [13,14]. Several studies showed that in patients with active UC, the administration of butyrate in association to standard therapy led to a significant improvement of inflammatory parameters [15,16,17,18,19]. Furthermore, we recently showed that add-on therapy with microencapsulated-sodium-butyrate in patients with UC was effective in maintenance of clinical remission (83.3%) compared to those treated with mesalamine only (47.6%) [20]. 

Given the qualitative and quantitative alterations observed in gut microbiota composition of patients with UC, another therapeutic approach pursued in the last years is the administration of probiotics and/or prebiotics [21]; the restoration of microbiota homeostasis may lead to the modulation of the local inflammatory response in the colon, and consequently, to the modification of the disease course. To date, the only approved alternative to 5-ASA in maintaining UC remission is *Escherichia coli* Nissle 1917 while no other probiotics/prebiotics are currently endorsed by international guidelines, apart from the probiotic mixture VSL#3 in pouchitis [22]. 

The aim of this study was to evaluate the efficacy of a gastro-resistant with colonic release patented preparation based on calcium butyrate, *Bifidobacterium bifidum*, *Bifidobacterium lactis*, and fructooligosaccharides (FOS) (FEEDColon^®^, Princeps, Cuneo, Italy) in maintaining remission, and improving subjective symptoms and inflammatory indices in patients with UC receiving 5-ASA therapy.

## 2. Materials and Methods

### 2.1. Study Design and Patients

This observational, single centre, prospective cohort study was conducted at the outpatient clinic of the Unit of Gastroenterology of “Città della Salute e della Scienza di Torino–Molinette” Hospital, Turin, Italy, between January 2018 and February 2019. 

Inclusion criteria were: age ≥18 years, histological diagnosis of UC according to the European Crohn’s and Colitis Organisation (ECCO) guidelines [22], UC in clinical remission under 5-ASA treatment, and at least one altered inflammatory index (C-reactive protein (CRP), erythrocyte sedimentation rate (ESR), faecal calprotectin (FC)). 

Exclusion criteria were: clinically active UC defined by a Mayo Partial Score (MPS) ≥ 3 [23], disease extension limited to the rectum, previous intestinal surgery (resections, colectomy), topical or systemic steroid therapy, treatment with antibiotics or supplementation with other probiotics/prebiotics in the 30 days before enrolment.

All patients received standard therapy with oral 5-ASA at a dose of 2400 mg/day during the 12 months follow-up (FU). At enrolment, patients were randomly assigned (1:1) to standard 5-ASA treatment group (Controls) and to 5-ASA plus FEEDColon^®^ supplementation (Cases). The latter were administered with FEEDColon^®^ at a dose of 2 tablets/day (1 tablet every 12 h, at breakfast and dinner) in addition to 5-ASA. All the patients underwent clinical assessment at baseline (T0), at 6-month FU (T1), and 12-month FU (T2). At each scheduled visit, we collected clinical data (disease activity by MPS, quality of life, abdominal pain, stool consistency) and biochemical parameters, including CRP, ESR, and FC. 

Disease-related quality of life was evaluated through the self-administration of the Short Inflammatory Bowel Disease Questionnaire (SIBDQ) [24]; the questionnaire evaluates patients’ physical, social, and emotional status by providing a score from 10 to 70, where 10 represents the minimum score obtainable by the patient and 70 the maximum. Abdominal pain was evaluated using a visual analogue scale (VAS) [25]; the scale ranges from 0 to 10, with 0 indicating absence of pain and 10 indicating the most intense pain imaginable by the patient. Finally, stool consistency was assessed using the Bristol stool scale (ranging from 1 to 7), where 1 indicates hard and goat stools (severe constipation), 2 hard sausage-shaped stools (moderate constipation), 3 soft stools with broken sausage (normal condition), 4 soft, smooth sausage stools (normal condition), 5 semi-balled stools, (reduced stool consistency), 6 semi-formed stools of more watery consistency (moderate diarrheal state), 7 completely watery stools (severe diarrheal condition) [26].

### 2.2. Outcomes

The primary outcome of the study was the maintenance of disease remission after 1 year of FEEDColon^®^ supplementation. Treatment was defined as successful for MPS ≤ 2 and FC < 250 µg/g at T2, without the need to modify therapy during the FU.

The secondary outcomes were the improvement of subjective symptoms (quality of life, abdominal pain, and stool consistency) and inflammatory parameters (CRP, ESR, FC) at 6- and 12-month FU.

### 2.3. Sample Size Estimation and Power Analysis

Based on our previous results and those by Miele and colleagues [20,27], we estimated that a proportion of patients maintaining remission of 81% in the 5-ASA plus FEEDColon^®^ group while 38% in the 5-ASA only group. Given a type I error (alpha) set at 0.05, a type II error (beta) set at 0.20, and a ratio of sample size of 1, the required sample size was 20 patients in the first group and 20 in the second group.

### 2.4. Statistical Analysis

Continuous variables were reported as median and interquartile range (IQR) or mean ± standard deviation (SD). The distribution of continuous variable was assessed by D’Agostino-Pearson test. Categorical variables were reported as number (*n*) and percentage (%). Comparison of continuous variables between independent groups was performed by Mann–Whitney test or unpaired Student’s *t* test, while comparison between paired measurements was performed by Wilcoxon test or paired Student’s *t* test. Friedman test or repeated measures analysis of variance (ANOVA) were used for kinetics analysis. Regarding dichotomous categorical variable, Fisher’s Exact test and McNemar test were performed to analyse unpaired or paired dichotomous categorical variables, respectively. Chi-squared (χ^2^) test was used to analyse contingency tables with more than 2 rows and/or columns.

All statistical analyses were performed by using MedCalc^®^ v.18.9.1 (MedCalc Software Ltd., Ostend, Belgium) and a *p* value ≤ 0.05 was considered statistically significant.

## 3. Results

Forty-two patients with UC in remission were included in the study; 21 underwent standard mesalamine treatment plus FEEDColon^®^ supplementation (Cases) while 21 standard mesalamine alone (Controls). Baseline demographic, clinical and biochemical characteristics of the patients enrolled are reported in Table 1. 

Overall, median age was 49 (35–58) years, with a higher prevalence of males (*n* = 25; 60%). Following randomization, no differences were observed at baseline concerning demographic, clinical, and biochemical characteristics. 

**Figure 1:**
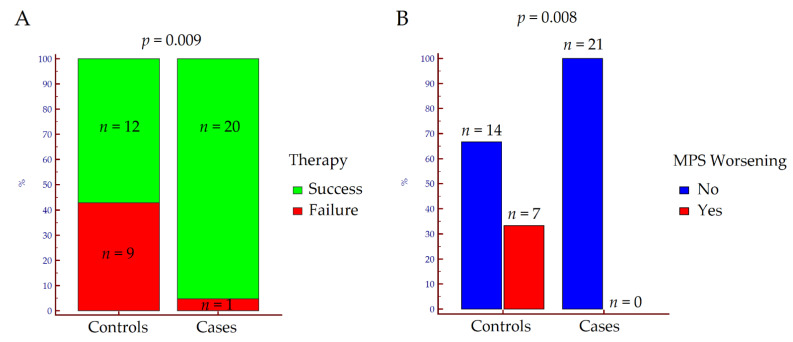
Rates of therapeutic success (**A**) and Mayo Partial Score worsening (**B**) at T2 according to treatment. *p* values were calculated by Fisher’s exact test. Abbreviations: MPS, Mayo Partial Score; *n*, number.

Regarding the primary outcome, therapeutic success assessed at T2 was achieved by 32 out of 42 (76%) of the patients enrolled; 20 out of 21 (95%) among those treated with 5-ASA + FeedColon^®^, and 12 out of 21 (57%) among those treated with 5-ASA only (*p* = 0.009) (Figure 1A). In the former, all patients (*n* = 21; 100%) maintained or reduced the MPS value, while in the latter, only 14 (66%) patients did not experience an MPS worsening (*p* = 0.008) at the end of FU (Figure 1B). 

**Figure 2:**
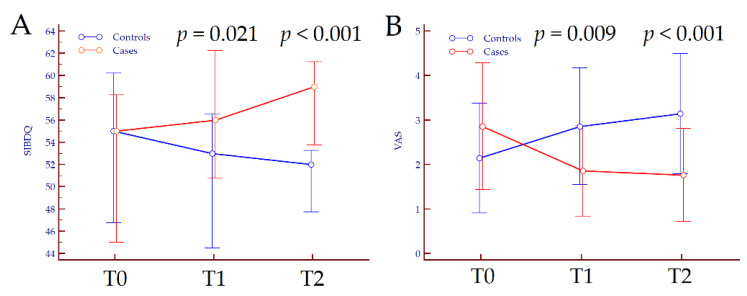
Variation of Short Inflammatory Bowel Disease Questionnaire score (**A**) and visual analogue score (**B**) from T0 to T2 according to treatment. *p* values refer to the comparison between groups at each time-point and were calculated by Mann–Whitney. Abbreviations: SIBDQ, Short Inflammatory Bowel Disease Questionnaire; T, timepoint; VAS, visual analogue scale.

**Figure 3:**
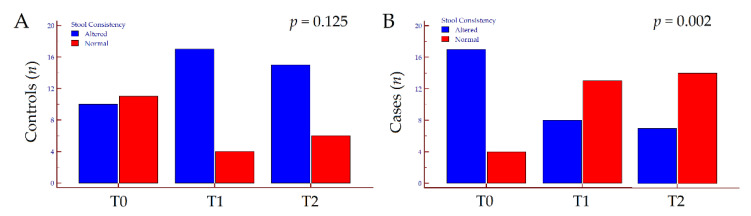
Variation of stool consistency in patients treated with mesalamine only (**A**) and those treated with mesalamine plus FEEDColon^®^ (**B**) from T0 to T2 according to treatment. *p* values were calculated by McNemar test. Abbreviations: *n*, number; T, timepoint.

Concerning secondary outcomes, quality of life assessed by SIBDQ did not vary in the overall population from T0 to T2 (*p* = 0.745). Conversely, we observed opposite trends of variation according to treatment; in patients treated with 5-ASA only, SIBDQ slightly diminished from 55 (47–60) at T0 to 52 (48–53) at T2 (*p* = 0.040), while in patients supplemented with FEEDColon^®^, SIBDIQ improved from 55 (45–58) at T0 to 59 (54–60) at T2 (*p* < 0.001). Similarly, VAS did not vary in the overall population from T0 to T2 (*p* = 0.763), whereas worsened in patients treated with 5-ASA only (*p* < 0.001) and ameliorated in those treated with 5-ASA plus FEEDColon^®^ (*p* < 0.001). Finally, the latter improved stool consistency at the end of FU (*p* = 0.002), while no significant changes were observed in patients treated with 5-ASA only (*p* = 0.125) (Table S1). The kinetics of subjective symptoms and the variation in stool consistency from T0 to T2 are depicted in Figure 2 and Figure 3.

**Figure 4:**
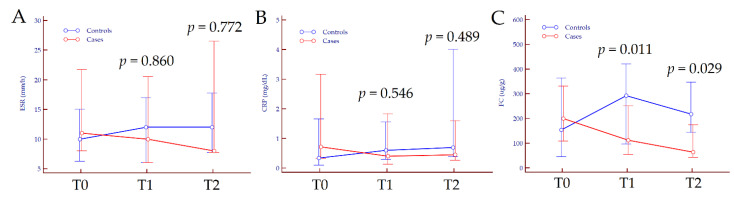
Variation of erythrocyte sedimentation rate (**A**), C-reactive protein (**B**) and faecal calprotectin values (**C**) from T0 to T2 according to treatment. *p* values refer to the comparison between groups at each time-point and were calculated by Mann–Whitney test. Abbreviations: CRP, C-reactive protein; ESR, erythrocyte sedimentation rate; FC, faecal calprotectin; T, timepoint.

Data on the modification of inflammatory indices (ESR, CRP, and FC) from baseline to end of FU are reported in Table S2. No significant variation was observed regarding ESR values, neither in patients treated with 5-ASA only nor in those treated with combination therapy (*p* = 0.269 and *p* = 0.107, respectively); CRP values slightly increased in the former while decreased in the latter (*p* = 0.020 and *p* = 0.013, respectively). Concerning FC levels, we observed a significant increase from T0 to T2 in patients treated with 5-ASA only, from baseline values of 154 (45–364) µg to 218 (144–348) µg/g at the end of FU (*p* = 0.003); in patients treated with 5-ASA plus FEEDColon^®^, FC values significantly diminished from baseline (200 (108–331) µg/g) to the end of FU (64 (43–175) µg/g) (*p* = 0.003). Overall, 31 out of 42 (74%) patients showed FC values < 250 µg/g after 12 months of therapy: 11 out of 21 (52%) in the 5-ASA treatment arm and 20 out of 21 (95%) in the 5-ASA plus FEEDColon^®^ group (*p* = 0.004). The kinetics of ESR, CRP and FC from T0 to T2 are depicted in Figure 4.

## 4. Discussion

In the present prospective study, we compared the efficacy in maintaining remission and improving subjective symptoms and inflammatory indices in a group of UC patients treated with 5-ASA only, compared to another group of UC patients treated with combination therapy of 5-ASA plus FEEDColon^®^, a patented preparation based on calcium butyrate, *Bifidobacterium bifidum*, *Bifidobacterium lactis*, and FOS. We observed that most patients treated with combination therapy (95%) maintained remission compared to the 57% of those treated with 5-ASA only. Furthermore, patients on combination therapy achieved a significant improvement of subjective symptoms, including quality of life, abdominal pain, and stool consistency as compared to those treated with standard maintenance therapy. The latter did not improve, or even worsened, the symptoms from baseline to 12-month of FU. Finally, FC values significantly diminished in patients treated with combination therapy, while improved in those treated with 5-ASA only.

The yearly relapse rate of patients with UC in remission has been estimated between 12% and 58% [28,29,30]. Previous studies showed that the supplementation of SCFA, probiotics/prebiotics in addition to standard therapy reduced the risk of relapse [31]. Miele and colleagues observed that 11 out of 14 (79%) patients treated with probiotics plus specific IBD therapy, maintained disease remission compared to 4 of 15 (27%) of those treated with placebo and IBD therapy (*p* = 0.014) [27]. However, a recent Cochrane meta-analysis did not find out a clear difference in clinical remission maintenance when probiotics combined with 5-ASA was compared with 5-ASA alone (risk ratio = 1.05, 95% CI 0.89–1.24) [32]. In our previous study, we investigated the effect of oral microencapsulated sodium butyrate (but without probiotics/prebiotics) administration in maintaining remission in patients with UC [20]; we observed that the majority of patients (83.3%) receiving microencapsulated sodium butyrate in combination to 5-ASA maintained clinical remission compared to the 47.6% of patients who underwent standard therapy with 5-ASA only (*p* = 0.022) [20]. Conversely, different studies comparing the relapse rate of UC patients treated with probiotics/prebiotics only vs. placebo, failed to demonstrate an efficacy in maintaining a log-term remission [33,34]. Taken together, it appears that butyrate rather than probiotics/prebiotics supplementation is the major determinant of the improved remission rates; conversely, we can hypothesize a synergistic beneficial effect of probiotics/prebiotics due to simultaneous colonic release. Overall, we can cautiously speculate that SCFA/probiotics/prebiotics could be a valid therapeutic approach to improve remission rate in patients with UC only if administered in combination with standard IBD treatment.

Concerning secondary outcomes, we observed an overall improvement of subjective symptoms (quality of life, abdominal pain, stool consistency) in patients treated with combination therapy as compared to those treated with 5-ASA only. Consistently, in the former group of patients, FC values diminished from baseline to 6-month FU, and further reduced at last FU, while distinctly improved in the latter group. FC is the most sensitive and specific intestinal inflammatory biomarker, widely used for the prediction of the clinical course in patients with UC [35,36,37]. It has been reported that FC values > 130 g/Kg were significantly associated to higher risk of relapse in UC patients (59% vs. 21%, *p* < 0.001) [38]. 

We recognize that the present study has limitations. In particular, the study was not double-blind; both patients and caregivers were aware of treatment allocation. Therefore, we cannot fully exclude any possible bias affecting subjective criteria of response. However, these results agreed with the improvement of objective criteria, particularly with FC, supporting the efficacy of FEEDColon^®^ add-on therapy in patients with UC in clinical remission. Another limitation may be represented by the lack of colonoscopy to assess endoscopic remission. However, in accordance with previous findings [1,39,40], we opted to measure FC (cut-off 250 µg/g) as a widely recognized biomarker of endoscopic remission.

## 5. Conclusions

In conclusion, the supplementation with FEEDColon^®^ to standard therapy with 5-ASA resulted superior to 5-ASA alone to maintain disease remission in patients with UC. Furthermore, combination therapy appeared to be more effective in improving subjective symptoms and inflammatory markers, especially FC. Further multicentre, placebo-controlled, double-blind clinical trials are needed to validate our results on a larger population of patients with UC in remission.

## Figures and Tables

**Table 1:** Baseline characteristics of the overall study population and according to treatment regimen.

Characteristics	Overall	Cases	Controls	*p* Value
Patients, *n* (%)	42 (100%)	21 (50%)	21 (50%)	
Age (years), median (IQR)	49 (35–58)	50 (33–65)	48 (40–57)	0.706
Gender (M/F)	25/17	11/10	14/7	0.530
MPS, *n* (%)				
0	7 (17%)	2 (9%)	5 (24%)	0.390
1	20 (47%)	10 (48%)	10 (48%)	
2	15 (36%)	9 (43%)	6 (28%)	
SIBDQ, median (IQR)	55 (46–59)	55 (45–58)	55 (47–60)	0.546
VAS, mean ± SD	2.5 ± 1.4	2.9 ± 1.4	2.1 ± 1.2	0.107
Bristol stool scale, *n* (%)				
1	0	0	0	0.151
2	1 (2%)	1 (5%)	0	
3	4 (10%)	2 (10%)	2 (10%)	
4	9 (21%)	2 (10%)	7 (33%)	
5	18 (43%)	7 (33%)	11 (52%)	
6	8 (19%)	8 (38%)	0	
7	2 (5%)	1 (5%)	1 (5%)	
ESR (mm/h), median (IQR)	10 (8–17)	11 (8–22)	10 (6–15)	0.203
CRP (mg/dL), median (IQR)	0.4 (0.3–1.8)	0.7 (0.3–3.2)	0.4 (0.1–1.7)	0.068
FC (µg/g), median (IQR)	174 (90–350)	208 (108–331)	154 (45–364)	0.308

*p* values were calculated by Mann–Whitney test or Student’s *t* test for continuous variables, while by χ^2^ test for categorical data. Abbreviations: CRP, C-reactive protein; ESR, erythrocyte sedimentation rate; F, female; FC, faecal calprotectin; IQR, interquartile range; M, male; MPS, Mayo partial score; *n*, number; SD, standard deviation; SIBDQ, Short Inflammatory Bowel Disease Questionnaire; VAS, visual analogue scale.

## Data Availability

The data presented in this study are available upon request from the corresponding author.

## Supplementary Materials

The following are available online at https://www.mdpi.com/article/10.3390/jcm10214961/s1, Table S1: Quality of life, abdominal pain, and stool consistency from T0 to T2 in the overall population of patients with UC and according to therapy, Table S2: Erythrocyte sedimentation rate, C-reactive protein, and faecal calprotectin values from T0 to T2 in the overall population of patients with UC and according to therapy.
