# Supplementary material for: Efficacy of a Preparation Based on Calcium Butyrate, Bifidobacterium bifidum, Bifidobacterium lactis, and Fructooligosaccharides in the Prevention of Relapse in Ulcerative Colitis: A Prospective Observational Study
Source: J Clin Med. 2021 Oct 26;10(21):4961. doi: 10.3390/jcm10214961 (PMC8585056; doi:10.3390/jcm10214961)
Supplement: Supplementary file 1 [file jcm-10-04961-s001.zip › jcm-1411828-supplementary.pdf]

Supplementary Material

Table S1. Quality of life, abdominal pain, and stool consistency from T0 to T2 in the overall population of patients with UC and according to therapy.

Parameters	*n*	T0	T1	T2	*p* Value
SIBDQ score, median (IQR)					
Overall population	42	55 (46–59)	54 (48–59)	54 (50–59)	0.745
5-ASA	21	55 (47–60)	53 (45–57)	52 (48–53)	0.040
5-ASA + FEEDColon®	21	55 (45–58)	56 (51–62)	59 (54–60)	<0.001
VAS, median (IQR)					
Overall population	42	2.5 ± 1.4	2.4 ± 1.3	2.5 ± 1.4	0.763
5-ASA	21	2.1 ± 1.2	2.9 ± 1.3	3.1 ± 1.4	<0.001
5-ASA + FEEDColon®	21	2.9 ± 1.4	1.9 ± 1.0	1.8 ± 1.0	<0.001
Normal stool consistency, *n* (%)*					
Overall population	42	15 (36%)	17 (41%)	20 (48%)	0.332
5-ASA	21	11 (52%)	4 (19%)	6 (29%)	0.125
5-ASA + FEEDColon®	21	4 (19%)	13 (62%)	14 (67%)	0.002

* Defined according to Bristol Stool Scale 3 and 4. *p* values were calculated by Friedman test/ ANOVA or McNemar test for continuous and categorical variables, respectively. Abbreviations: 5-ASA, mesalamine; IQR, interquartile range; *n*, number; SIBDQ, Short Inflammatory Bowel Disease Questionnaire; T, timepoint; VAS, visual analogue scale.

Table S2. Erythrocyte sedimentation rate, C-reactive protein, and fecal calprotectin values from T0 to T2 in the overall population of patients with UC and according to therapy.

Parameters	*n*	T0	T1	T2	*p* Value
ESR (mm/h), median (IQR)					
Overall population	42	10 (8–17)	11 (6–17)	11 (8–22)	0.433
5-ASA	21	10 (6–15)	12 (6–17)	12 (8–18)	0.269
5-ASA + FEEDColon®	21	11 (8–22)	10 (6–21)	8 (8–27)	0.107
CRP (mg/dL), median (IQR)					
Overall population	42	0.4 (0.3–1.8)	0.5 (0.2–1.4)	0.6 (0.3–3.4)	0.266
5-ASA	21	0.3 (0.1–1.7)	0.6 (0.3–1.6)	0.7 (0.4–4.0)	0.020
5-ASA + FEEDColon®	21	0.7 (0.3–3.2)	0.4 (0.1–1.8)	0.5 (0.3–1.6)	0.013
FC (μg/g), median (IQR)					
Overall population	42	174 (90–350)	176 (58–312)	149 (58–281)	0.281
5-ASA	21	154 (45–364)	293 (96–421)	218 (144–348)	0.003
5-ASA + FEEDColon®	21	200 (108–331)	112 (55–251)	64 (43–175)	0.003

p values were calculated by Friedman test. Abbreviations: 5-ASA, mesalamine; CRP, C-reactive protein; ESR, erythrocyte sedimentation rate; FC, fecal calprotectin; IQR, interquartile range; *n*, number.
